# Unexpected detection of SARS-CoV-2 antibodies in the prepandemic period in Italy

**DOI:** 10.1177/0300891620974755

**Published:** 2020-11-11

**Authors:** Giovanni Apolone, Emanuele Montomoli, Alessandro Manenti, Mattia Boeri, Federica Sabia, Inesa Hyseni, Livia Mazzini, Donata Martinuzzi, Laura Cantone, Gianluca Milanese, Stefano Sestini, Paola Suatoni, Alfonso Marchianò, Valentina Bollati, Gabriella Sozzi, Ugo Pastorino

**Affiliations:** 1 Fondazione IRCCS Istituto Nazionale Tumori, Milan, Italy; 2 Faculty of Medicine and Surgery, University of Siena, Siena, Italy; 3 VisMederi Srl, Siena, Italy; 4 VisMederi Research Srl, Siena, Italy; 5 EPIGET–Epidemiology, Epigenetics and Toxicology Lab, University of Milan, Milan, Italy; 6 Radiology, Department of Medicine and Surgery, University of Parma, Parma, Italy

**Keywords:** Screening, COVID-19, SARS-CoV-2 antibodies

## Abstract

There are no robust data on the real onset of severe acute respiratory syndrome coronavirus 2 (SARS-CoV-2) infection and spread in the prepandemic period worldwide. We investigated the presence of SARS-CoV-2 receptor-binding domain (RBD)–specific antibodies in blood samples of 959 asymptomatic individuals enrolled in a prospective lung cancer screening trial between September 2019 and March 2020 to track the date of onset, frequency, and temporal and geographic variations across the Italian regions. SARS-CoV-2 RBD-specific antibodies were detected in 111 of 959 (11.6%) individuals, starting from September 2019 (14%), with a cluster of positive cases (>30%) in the second week of February 2020 and the highest number (53.2%) in Lombardy. This study shows an unexpected very early circulation of SARS-CoV-2 among asymptomatic individuals in Italy several months before the first patient was identified, and clarifies the onset and spread of the coronavirus disease 2019 (COVID-19) pandemic. Finding SARS-CoV-2 antibodies in asymptomatic people before the COVID-19 outbreak in Italy may reshape the history of pandemic.

At the end of December 2019, the novel severe acute respiratory syndrome coronavirus 2 (SARS-CoV-2) causing serious pneumonia was identified in Wuhan, Hubei Province, China.^1^ The coronavirus disease 2019 (COVID-19) viral disease rapidly spread worldwide, and the World Health Organization declared pandemic status in March 2020 (www.who.int).

Italy’s first two cases of COVID-19 disease were recorded on January 30, 2020, when two tourists from China tested positive for SARS-CoV-2 in Rome. The first laboratory-confirmed Italian COVID-19 case was identified in Lombardy on February 20, 2020, in a 38-year-old man who had no history of possible contacts with positive cases in Italy or abroad. Within a few days, additional cases of COVID-19 and critically ill patients were recorded in the surrounding area. Soon several cases were identified in other Italian regions, mostly in the northern area. Lockdowns were first applied in 2 critical areas of Lombardy and Veneto and were rapidly enforced regionally and nationwide starting on March 8.

On the basis of the first case identification, it was hypothesized that the virus had been circulating in Italy since January 2020. However, the rapid spread, the large number of patients requiring hospital admission and treatment in intensive care units, as well as the duration of the pandemic suggest that the arrival of the virus and its circulation in Italy in a less symptomatic form could be anticipated by several months.

Serologic assays can be used to investigate antibody responses against SARS-CoV-2 infection and assess its real prevalence.^2^ Anti-SARS-CoV-2 antibody response analyses in patients with COVID-19 showed that within 13 days after symptom onset, seroconversion of antiviral immunoglobulin G (IgG) or immunoglobulin M (IgM) was present in almost 100% of patients.^3^

To test the hypothesis of early circulation of the virus in Italy, we investigated the frequency, timing, and geographic distribution of SARS-CoV-2 exposure in a series of 959 asymptomatic individuals, using proprietary SARS-CoV-2 binding and neutralizing antibodies on the plasma samples repository. The population was enrolled from September 2019 to March 2020 through the SMILE trial (Screening and Multiple Intervention on Lung Epidemics; ClinicalTrials.gov Identifier: NCT03654105), a prospective lung cancer screening study using low-dose computed tomography and blood biomarkers, with the approval of our institutional review board and ethics committee. All eligible participants provided written informed consent.

A receptor-binding domain (RBD)–specific enzyme-linked immunosorbent assay (ELISA) test was performed and qualified as reported by Mazzini and colleagues.^4^ A qualitative microneutralization assay was performed as previously reported.^5^ Details can be found in the Supplementary Material.

SMILE cohort characteristics are shown in the Supplementary Table S1. In summary, 397 patients (41.4%) were women, 63.2% were 55–65 years old, 76.8% were current smokers, and 92.9% had smoked ⩾30 pack-years. Overall, 111 of 959 (11.6%) plasma samples showed SARS-CoV-2 RBD-specific antibodies (IgM, IgG, or both). In particular, IgM antibodies were detected in 97 (10.1%) patients; IgG antibodies were found in 16 (1.7%). All the patients were asymptomatic at the time of blood sample collection.

Table 1 reports anti-SARS-CoV-2 RBD antibody detection according to the time of sample collection in Italy. In the first 2 months, September–October 2019, 23/162 (14.2%) patients in September and 27/166 (16.3%) in October displayed IgG or IgM antibodies, or both. The first positive sample (IgM-positive) was recorded on September 3 in the Veneto region, followed by a case in Emilia Romagna (September 4), a case in Liguria (September 5), two cases in Lombardy (Milano Province; September 9), and one in Lazio (Roma; September 11). By the end of September, 13 of the 23 (56.5%) positive samples were recorded in Lombardy, three in Veneto, two in Piedmont, and one each in Emilia Romagna, Liguria, Lazio, Campania, and Friuli. A similar time distribution was observed when considering Lombardy alone (Supplementary Table S2).

**Table 1.** Severe acute respiratory syndrome coronavirus 2 (SARS-CoV-2) receptor-binding domain antibodies detection according to time of sample collection in all regions.

Month	Patients	IgG+, n (%)	IgM+, n (%)	IgG+ and/or IgM+, n (%)
Total	959	16 (1.7)	97 (10.1)	111 (11.6)
September	162	3 (1.9)	20 (12.4)	23 (14.2)
October	166	4 (2.4)	23 (13.9)	27 (16.3)
November	273	3 (1.1)	23 (8.4)	26 (9.5)
December	147	3 (2.0)	10 (6.8)	11 (7.5)
January	106	1 (0.9)	2 (1.9)	3 (2.8)
February	105	2 (1.9)	19 (18.1)	21 (20.0)

IgG: immunoglobulin G; IgM: immunoglobulin M.

The diagram in Figure 1 illustrates the temporal variation in positive samples from September 2019 to February 2020. Notably, two peaks of positivity for anti-SARS-CoV-2 RBD antibodies were visible: the first one started at the end of September, reaching 18% and 17% of IgM-positive cases in the second and third weeks of October, respectively. A second one occurred in February 2020, with a peak of over 30% of IgM-positive cases in the second week. Out of this cluster of 16 positive samples, 11 (68.7%) originated in Lombardy.

**Figure 1.**
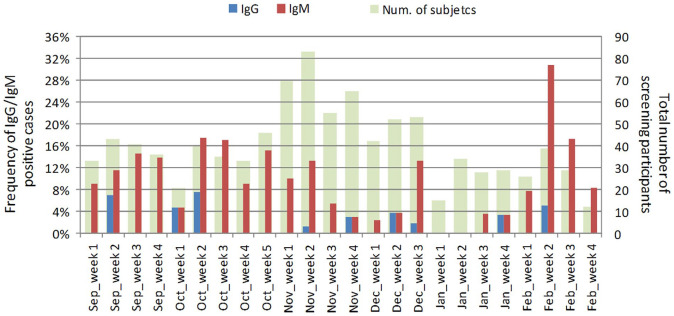
Frequency of immunoglobulin M (red columns) and immunoglobulin G (blue columns) receptor-binding domain (RBD)–positive cases in respect to the total number of screening participants (green columns) throughout the 24 weeks from September 2019 to February 2020.

The national distribution of the 959 recruited patients and of the 111 who tested positive for RBD-SARS-CoV-2 antibodies in comparison with the allocation of the patients with COVID-19 identified in Italy up to March 10 (last SMILE study recruitment date) is shown in Figure 2(A) and Supplementary Table S3. The 959 recruited patients came from all Italian regions, and at least one SARS-CoV-2–positive patient was detected in 13 regions. According to data collected from the website of the Italian Ministry of Health (www.salute.gov.it), Lombardy was the region most affected by the pandemic, with 5791/10,141 (57.1%) patients with COVID-19, and showed the highest number of recruited patients at 491/959 (51.2%). Considering the 111 positive cases, 59 (53.2%) were in residents of Lombardy, followed by Piedmont and Lazio (10 cases each, 9%); Emilia Romagna (7 cases, 6.3%); Tuscany and Veneto (6 cases each, 5.4%); Liguria (4 cases, 3.6%); Campania, Friuli, and Puglia (2 cases each, 1.8%); and Sicily, Valle d’ Aosta, and Sardinia (1 case each, 0.9%).

**Figure 2.**
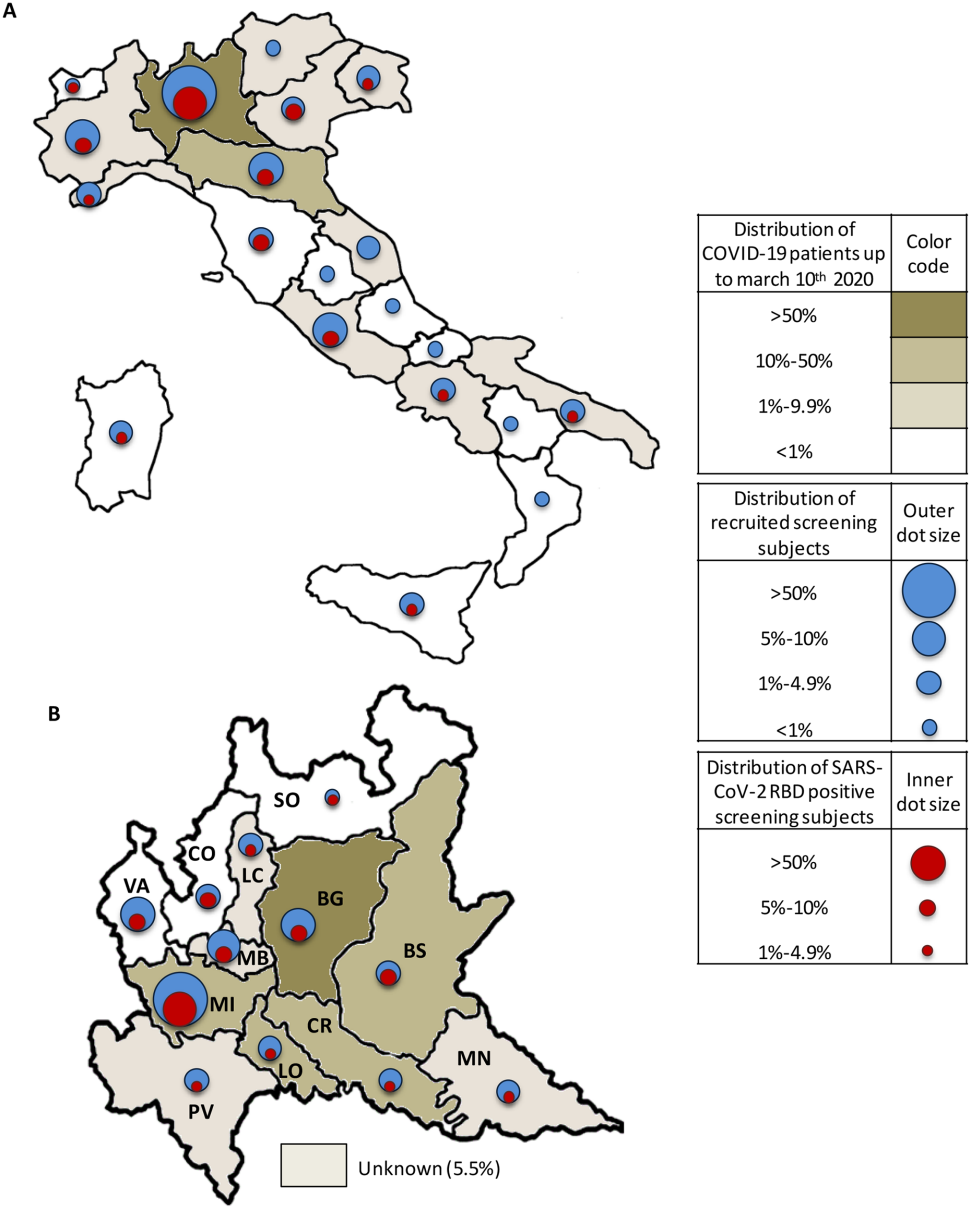
Comparison of the distribution of patients with coronavirus disease 2019 (COVID-19) identified up to March 10, 2020, according to data of the Italian Ministry of Health (www.salute.gov.it), with the distribution of recruited screening subjects (blue dots) and SARS-CoV-2 receptor-binding domain (RBD)–positive screening subjects (red dots) of the SMILE trial (Screening and Multiple Intervention on Lung Epidemics). The national distribution includes 10,149 patients with COVID-19, the 959 recruited screening subjects, and the 111 SARS-CoV-2 RBD-positive screening subjects across the 20 Italian regions **(A)**. The regional distribution includes 5791 patients with COVID-19, the 491 recruited screening subjects, and the 59 SARS-CoV-2 RBD-positive screening subjects across the 12 provinces of Lombardy **(B)**.

Figure 2B and Supplementary Table S4 show distribution across the 12 Lombardy provinces of the 491 recruited patients and of the 59 patients testing positive for anti-SARS-CoV-2 RBD antibodies in comparison with the allocation of the 5791 patients with COVID-19 identified up to March 10 in the same region (www.salute.gov.it). Overall, 30 positive patients were detected in Milan Province and 29 in the other provinces analyzed (Monza: 6, Como: 5, Bergamo: 4, Brescia: 3, Varese: 3, Lecco: 2, Pavia: 2, Mantova: 1, Lodi: 1, Cremona: 1, and Sondrio: 1). Therefore the geographic distribution and timing of the SARS-CoV-2–positive individuals identified in our study closely mirrors the incidence of COVID-19 officially registered in Italy.

Evaluation of anti-SARS-CoV-2 functional neutralizing antibodies (NAbs) was performed for all 111 SARS-CoV-2 RBD-positive samples using a cytopathic effect (CPE)–based live virus microneutralization assay in a high-containment biosafety level 2 laboratory. Six of the 111 SARS-CoV-2 RBD-positive patients were positive in the qualitative CPE-based microneutralization test. Of these, four samples were collected in October (two on the 7th, one each on the 8th and the 21st), one in November, and one in February. Three of the positive NAb samples were from Lombardy, one from Lazio, one from Tuscany, and one from Valle d’Aosta. The presence of functional anti-SARS-CoV-2 NAbs at the beginning of October 2019 further supports the early unnoticed circulation of the virus in Italy, particularly in Lombardy.

At the end of December 2019, COVID-19 appeared in Wuhan City, China. As of September 12, 2020, 37,584,742 COVID-19 cases were confirmed worldwide, with more than 1 million deaths (https://coronavirus.jhu.edu/map.html). In Italy, the first case was identified in Lombardy on February 20, and the first death attributed to COVID-19 occurred in a 77-year-old retiree living in a small town in the Veneto region. In mid-September 2020, the number of official cases in Italy reached approximately 300,000, with over 35,000 deaths (www.salute.gov.it), but it is likely that these numbers do not reflect the actual onset and epidemiology of SARS-CoV-2 in Italy.

Two phenomena need to be highlighted and discussed. The first concerns the underestimation of the prevalence of cases. Regional and national health authorities, after an attempt to identify cases and suspects early and trace all the potential contacts, soon abandoned this strategy as unsustainable and concentrated on the identification strategy, with swabs and serology, on symptomatic cases only. As a consequence, an underestimation of overall COVID-19 cases was created, and a selection bias was introduced, with an overestimation of the mortality rate. Well-designed serosurveys in selected subpopulations with specific risk groups have provided valuable epidemiologic information. The prevalence of SARS-CoV-2 infection was tested in 8285 health care workers of the main hospitals of the Veneto Region between February 22 and May 29, 2020. By measuring specific antibodies, an overall prevalence of 4.6% was observed. Although detectable antibodies were found in all workers who developed severe COVID-19 infection (100%), lower seropositivity was found in those with mild disease (83%), and the lowest prevalence (58%) was observed in asymptomatic individuals.^6^ Between May 25 and July 15, the Italian Ministry of Health accomplished a large SARS-CoV-2 seroprevalence study in a representative sample of 64,660 individuals. A global prevalence rate of 2.5% was reported, with a peak in the Lombardy region (7.5%) and in particular in Bergamo Province (24%) (www.salute.gov.it). As a consequence, the true number of Italians who had been in contact with the virus would be approximately 1.5 million, many of whom were asymptomatic, an estimate almost 5 times higher than the official figures reported.

The second concern regards the onset of the epidemic, which is likely to have preceded the identification of the first case, probably in the last part of 2019. Since November–December 2019, many general practitioners began reporting the appearance of severe respiratory symptoms in elderly and frail people with atypical bilateral bronchitis, which was attributed, in the absence of news about the new virus, to aggressive forms of seasonal influenza. One investigation on SARS-CoV-2 seroprevalence in healthy blood donors has been performed in one of the two initial lockdown areas in northern Italy.^7^ In a group of 300 stored plasma samples, 5 samples collected between the 12th and 17th of February exhibited evidence of anti-SARS-CoV-2 NAbs. Moreover, a phylogenetic analysis of the SARS-CoV-2 genomes isolated from 3 Lombardy patients involved in the first COVID-19 outbreak suggests that the common origin of the strains dates back several weeks before the first cases of COVID-19 pneumonia reported in China.^8^ Based on these findings, a prior unnoticed circulation of the virus among the Italian population could be hypothesized.

Given the rapid increase in symptomatic cases worldwide, a better understanding of the initial history and epidemiology of COVID-19 could improve the screening strategy and contain the effects of a possible second wave. Evidence from environmental monitoring showed that SARS-CoV-2 was already circulating in northern Italy at the end of 2019.^9^ Molecular analysis with reverse transcription polymerase chain reaction assays of 40 composite influent wastewater samples collected between October 2019 and February 2020 in three cities and regions in northern Italy (Milan/Lombardy, Turin/Piedmont, and Bologna/Emilia Romagna) showed the presence of viral RNA first occurring in sewage samples collected on December 18 in Milan and Turin. This study also indicates that SARS-CoV-2 was circulating in different geographic regions simultaneously, which agrees with our serologic findings.

At the international level, concordant evidence comes from two additional studies. A first article reported a case of a patient hospitalized for hemoptysis with no etiologic diagnosis in an intensive care unit in Paris, France, in December 2019.^10^ Retrospective molecular analysis on the stored nasopharyngeal swab confirmed the diagnosis of SARS-CoV-2 infection. A second study by Harvard University showed a relevant increase of hospital traffic in the Wuhan region, evaluated by satellite imagery, and COVID-19 symptoms–related queries in search engines, since autumn 2019.^11^ These findings suggest that the virus may have already been circulating at the time of the outbreak in several countries.

To our knowledge, there are no published data on antibody responses to SARS-CoV-2 in the prepandemic period in any countries in the world. Our study was carried out in a sample of asymptomatic individuals originating from all Italian regions. At least one SARS-CoV-2–positive individual was detected in 13 regions, and Lombardy had the highest number, mirroring the data from the national survey.

The first surge of positive cases was identified in September–October 2019. Evaluation of anti–SARS-CoV-2 functional NAbs identified positive samples in CPE-based microneutralization tests already collected in October 2019. Given the temporal delay between infection and antibody synthesis, these results indicate that the virus circulated in Italy well before the detection of the declared index patient in February 2020. In addition, most of the first antibody-positive individuals lived in regions where the pandemic started.

The serologic assay used in this study is an in-house designed RBD-based ELISA, namely, VM-IgG-RBD and VM-IgM-RBD, and is a proprietary assay developed by using spike glycoprotein (S-protein), which mediates binding to target cells through the interaction between the RBD and the human angiotensin-converting enzyme 2 (ACE2) receptor. The S-protein has been found to be highly immunogenic, and the RBD is considered the main SARS-CoV-2–specific target in the effort to elicit potent NAbs.^12^ In our preliminary study, an excellent correlation between the neutralization titer and the IgG, IgM, and immunoglobulin A ELISA response against the RBD of the S-protein was observed,^4^ confirming that the RBD-based ELISA can be used as a valid surrogate for neutralization. Therefore, the specificity of the assays used in the present study strongly supports our seroprevalence findings in a relevant number of asymptomatic individuals well before the overt pandemic period, with positive patients in September–October 2019.

Our results indicate that SARS-CoV-2 circulated in Italy earlier than the first official COVID-19 cases were diagnosed in Lombardy, even long before the first official reports from the Chinese authorities, casting new light on the onset and spread of the COVID-19 pandemic.

## Supplemental Material

Supplementary_Materials – Supplemental material for Unexpected detection of SARS-CoV-2 antibodies in the prepandemic period in Italy Click here for additional data file. Supplemental material, Supplementary_Materials for Unexpected detection of SARS-CoV-2 antibodies in the prepandemic period in Italy by Giovanni Apolone, Emanuele Montomoli, Alessandro Manenti, Mattia Boeri, Federica Sabia, Inesa Hyseni, Livia Mazzini, Donata Martinuzzi, Laura Cantone, Gianluca Milanese, Stefano Sestini, Paola Suatoni, Alfonso Marchianò, Valentina Bollati, Gabriella Sozzi and Ugo Pastorino in Tumori Journal
