# Supplementary material for: Unexpected detection of SARS-CoV-2 antibodies in the prepandemic period in Italy
Source: Tumori. 2020 Nov 11;107(5):446–51. doi: 10.1177/0300891620974755 (PMC8529295; doi:10.1177/0300891620974755)
Supplement: Supplementary_Materials – Supplemental material for Unexpected detection of SARS-CoV-2 antibodies in the prepandemic period in Italy [file Supplementary_Materials.docx]

Supplementary Materials for

Unexpected detection of SARS-CoV-2 antibodies in the pre-pandemic period in Italy

Giovanni Apolone^†^, Emanuele Montomoli^†^, Alessandro Manenti, Mattia Boeri, Federica Sabia, Inesa Hyseni, Livia Mazzini, Donata Martinuzzi, Laura Cantone, Gianluca Milanese, Stefano Sestini, Paola Suatoni, Alfonso Marchianò, Valentina Bollati, Gabriella Sozzi and Ugo Pastorino

Correspondence to: gabriella.sozzi@istitutotumori.mi.it

**This PDF file includes:**

Materials and Methods

Table S1

Table S2

Table S3

Table S4

Materials and Methods

SMILE cohort

The SMILE trial (Screening and Multiple Intervention on Lung Epidemics) is an ongoing randomized study testing the efficacy of early diagnosis with annual ultra-LDCT in lifelong smokers combined with multiple interventions to improve smoking cessation with oral cytisine and reduce chronic inflammatory status with low-dose acetylsalicylic acid (ASA), diet modification and increased physical activity. All volunteers underwent baseline LDCT, spirometry, carbon monoxide (CO) measurement, blood sample collection, and anthropometric and sociodemographic data collection. In addition, subjects allocated to each intervention arm received the specific treatment. The study was approved by our Institutional Review Board and Ethics Committee, and all eligible subjects provided written informed consent.

From July 2019 to March 2020, a total of 1114 volunteers were enrolled at the Istituto Nazionale Tumori of Milan and eventually randomized to each intervention or control arm through a factorial design. The trial amendment with SARS-CoV-2 implementation was approved by the Ethics Committee in April 2020. In the present analysis, we included 959 volunteers screened between September 2nd, 2019 and February 27th, 2020. Further information on study design, eligibility criteria and amendments are available at ClinicalTrials.gov Identifier: NCT03654105.

Live virus and antigen

Wild-type SARS-CoV-2 - strain 2019-nCoV/Italy INMI1 - was purchased from the European Virus Archive Global (EVAg, Spallanzani Institute, Rome) and propagated in VeroE6 (ATCC- CRL1586) cell culture. The purified HEK293 cell-derived receptor-binding domain (RBD) antigen was purchased from Sino Biological, code 40592-V08H.

Human monoclonal antibodies

Human anti-SARS-CoV-2 Spike (S1) IgM Antibody CR3022 (Native Antigen, Oxford, UK) and anti-Spike RBD (SARS-CoV-2/COVID-19) human monoclonal antibody (eEnzyme, Gaithersburg, MD, USA) were used as positive controls in an ELISA. Negative human serum (IgA/IgM/IgG) (Sigma-Aldrich, St. Louis, MO, USA) was used as a negative control.

Receptor-binding domain IgG and IgM enzyme-linked immunosorbent assay

An RBD-specific ELISA test was performed and qualified as already reported by Mazzini and colleagues (*4*). ELISA plates were coated with 1 µg/mL purified recombinant Spike RBD protein (Sino Biological, Beijing, China) in 0.05 M carbonate-bicarbonate solution and incubated overnight at +4°C. The day after, the plates were washed three times with 300 µl/well ELISA washing solution containing Tris-buffered saline (TBS)-0.05% Tween 20 and then blocked for 1 hour at 37°C with a solution of TBS containing 5% nonfat dry milk (NFDM) (Euroclone, Pero, Italy).

As a prevention and safety procedure, human serum samples were heat inactivated at 56°C for 1 hour and then diluted 1:100 (final dilution) in TBS-0.05% Tween 20 - 5% NFDM. The plates were washed three times as previously described, and 100 µl of each serum dilution was added to the coated plates and incubated for 1 hour at 37°C. Next, after the washing step, 100 µl/well goat anti-human IgG-Fc and goat anti-human IgM (µ-chain) HRP-conjugated antibodies were diluted 1:100,000 (Bethyl Laboratories, Montgomery, TX, USA), added to different plates and incubated at 37°C for 30 minutes. Following incubation, the plates were washed four times, and 100 µl/well 3,3′,5,5′-tetramethylbenzidine (TMB) substrate (Bethyl Laboratories, Montgomery, TX, USA) was added and incubated at room temperature in the dark for 20 minutes. The reaction was stopped by adding 100 µl of ELISA stop solution (Bethyl Laboratories, Montgomery, TX, USA) and read at 450 nm.

For each plate, two positive controls were added: an RBD-specific IgG1 monoclonal antibody (mAb) or IgM mAb and human positive serum from a COVID-19 convalescent patient. The cutoff value was established for each plate as 3 times the average of optical density (OD) values from blank wells (background-no addition of analyte). Samples with ODs under the cutoff value at the first (1:100) dilution were designated negative, and samples for which the ODs at the 1:100 dilution were above the cutoff value were designated positive. Borderline samples were defined where one replicate was under the cutoff and the other was above.

Qualitative microneutralization assay

A qualitative microneutralization (MN) assay was performed as previously reported (*5*). Briefly, serum samples were heat inactivated for 30 minutes at 56°C and then mixed in a 1:5 ratio with a SARS-CoV-2 viral solution containing 100 tissue culture infective dose 50% (TCID50) of virus (final volume, 120 µl). After one hour of incubation at 37°C and 5% CO2, 100 µl of each virus-supernatant mixture was added to the well of a 96-well plate containing 80% confluent Vero E6 cell monolayer. The plates were incubated for a total of three days at 37°C and 5% CO2 in a humidified atmosphere and then inspected for the presence/absence of cytopathic effects (CPEs) by means of an inverted optical microscope.

Statistical analysis

Descriptive statistics are reported as numbers and percentages. Homogeneity of variable distribution between selected groups was tested by the Chi-square test or Fisher's exact test, as appropriate. Trends between categorical variables were tested by the Cochran-Armitage trend test or Cochran-Mantel-Haenszel test, as appropriate. Analyses were performed using Statistical Analysis System Software (version 9.4; SAS Institute, Cary, NC, USA).

Table S1.

Characteristics of lung cancer screening participants.

| Characteristic | | | Number |
| --- | --- | --- | --- |
| TOTAL | |  | 959 |
| Sex | | |  |
|  | Female | | 397 (41.4%) |
|  | Male | | 562 (58.6%) |
| Age | | |  |
|  | <55 | | 75 (7.8%) |
|  | 55-65 | | 606 (63.2%) |
|  | ≥65 | | 278 (29.0%) |
| Smoking history | | |  |
|  | Former | | 223 (23.3%) |
|  | Current | | 736 (76.8%) |
| Pack-years | | |  |
|  | <30 | | 68 (7.1%) |
|  | ≥30 | | 891 (92.9%) |
| Body mass index | | |  |
|  | Underweight | | 13 (1.4%) |
|  | Normal weight | | 370 (38.5%) |
|  | Overweight | | 576 (60.1%) |

Table S2.

SARS-CoV-2 RBD-antibodies detection according to time of sample collection in Lombardy.

| Month | Total subjects | IgG | IgM | IgG and/or IgM |
| --- | --- | --- | --- | --- |
| TOTAL | 491 | 9 (1.8%) | 51 (10.3%) | 59 (12.0%) |
| September | 103 | 2 (1.9%) | 11 (10.7%) | 13 (12.6%) |
| October | 92 | 1 (1.1%) | 15 (16.3%) | 16 (17.4%) |
| November | 130 | 2 (1.5%) | 10 (7.7%) | 12 (9.2%) |
| December | 48 | 1 (2.1%) | 4 (8.3%) | 4 (8.3%) |
| January | 61 | 1 (1.6%) | 2 (3.3%) | 3 (4.9%) |
| February | 56 | 2 (3.5%) | 9 (16.1%) | 11 (19.6%) |

Table S3.

Distribution across the Italian regions of the 10,149 COVID-19 patients identified up to March 10th, 2020 (data of the Italian Ministry of Health), of the 959 screening subjects recruited for the SMILE study and of the 111 SARS-CoV-2 RBD-positive screening subjects.

| Italian Region | COVID-19 patients up to March 10^th^ | |  | Recruited Screening subjects | |  | SARS-CoV-2  RBD-positive screening subjects | |
| --- | --- | --- | --- | --- | --- | --- | --- | --- |
| TOTAL | 10,149 | 100% |  | 959 | 100% |  | 111 | 100% |
| Lombardia | 5791 | 57.1% |  | 491 | 51.2% |  | 59 | 53.2% |
| Lazio | 116 | 1.1% |  | 71 | 7.4% |  | 10 | 9.0% |
| Piemonte | 90 | 0.9% |  | 72 | 7.5% |  | 10 | 9.0% |
| Emilia-romagna | 1533 | 15.1% |  | 77 | 8.0% |  | 7 | 6.3% |
| Veneto | 856 | 8.4% |  | 36 | 3.8% |  | 6 | 5.4% |
| Toscana | 62 | 0.6% |  | 44 | 4.6% |  | 6 | 5.4% |
| Liguria | 141 | 1.4% |  | 19 | 2.0% |  | 4 | 3.6% |
| Puglia | 453 | 4.5% |  | 22 | 2.3% |  | 2 | 1.8% |
| Campania | 127 | 1.3% |  | 30 | 3.1% |  | 2 | 1.8% |
| Friuli venezia giulia | 116 | 1.1% |  | 13 | 1.4% |  | 2 | 1.8% |
| Sardegna | 59 | 0.6% |  | 13 | 1.4% |  | 1 | 0.9% |
| Sicilia | 20 | 0.2% |  | 25 | 2.6% |  | 1 | 0.9% |
| Valle d'aosta | 17 | 0.2% |  | 2 | 0.2% |  | 1 | 0.9% |
| Marche | 394 | 3.9% |  | 19 | 2.0% |  | 0 | 0.0% |
| Trentino alto adige | 264 | 2.6% |  | 4 | 0.4% |  | 0 | 0.0% |
| Abruzzo | 38 | 0.4% |  | 7 | 0.7% |  | 0 | 0.0% |
| Umbria | 37 | 0.4% |  | 5 | 0.5% |  | 0 | 0.0% |
| Molise | 15 | 0.1% |  | 3 | 0.3% |  | 0 | 0.0% |
| Calabria | 13 | 0.1% |  | 4 | 0.4% |  | 0 | 0.0% |
| Basilicata | 7 | 0.1% |  | 2 | 0.2% |  | 0 | 0.0% |

Table S4.

Distribution across the provinces of Lombardy of the 5,791 COVID-19 patients identified up to March 10th, 2020 (data of the Italian Ministry of Health), of the 491 screening subjects recruited in Lombardy for the SMILE study and of the 59 SARS-CoV-2 RBD-positive screening subjects.

| Province of Lombardy | COVID-19 patients up to March 10^th^ | |  | Recruited Screening subjects | |  | SARS-CoV-2  RBD-positive screening subjects | |
| --- | --- | --- | --- | --- | --- | --- | --- | --- |
| TOTAL | 5791 | 100% |  | 491 | 100% |  | 59 | 100% |
| Milan | 592 | 10.2% |  | 302 | 61.5% |  | 30 | 50.8% |
| Monza | 65 | 1.1% |  | 37 | 7.5% |  | 6 | 10.2% |
| Como | 46 | 0.8% |  | 17 | 3.5% |  | 5 | 8.5% |
| Bergamo | 1472 | 25.4% |  | 31 | 6.3% |  | 4 | 6.8% |
| Brescia | 790 | 13.6% |  | 24 | 4.9% |  | 3 | 5.1% |
| Varese | 50 | 0.9% |  | 28 | 5.7% |  | 3 | 5.1% |
| Pavia | 324 | 5.6% |  | 16 | 3.3% |  | 2 | 3.4% |
| Lecco | 89 | 1.5% |  | 17 | 3.5% |  | 2 | 3.4% |
| Lodi | 963 | 16.6% |  | 5 | 1.0% |  | 1 | 1.7% |
| Cremona | 957 | 16.5% |  | 5 | 1.0% |  | 1 | 1.7% |
| Mantova | 119 | 2.1% |  | 5 | 1.0% |  | 1 | 1.7% |
| Sondrio | 7 | 0.1% |  | 4 | 0.8% |  | 1 | 1.7% |
| Unknown | 317 | 5.5% |  | 0 | 0.0% |  | 0 | 0.0% |
